# Review of structured light in diffuse optical imaging

**DOI:** 10.1117/1.JBO.24.7.071602

**Published:** 2018-09-14

**Authors:** Joseph P. Angelo, Sez-Jade Chen, Marien Ochoa, Ulas Sunar, Sylvain Gioux, Xavier Intes

**Affiliations:** a National Institute of Standards and Technology, Sensor Science Division, Gaithersburg, Maryland, United States; b Rensselaer Polytechnic Institute, Department of Biomedical Engineering, Troy, New York, United States; c Wright State University, Department of Biomedical Industrial and Human Factor Engineering, Dayton, Ohio, United States; d University of Strasbourg, ICube Laboratory, Strasbourg, France

**Keywords:** compressed sensing, diffusion, imaging, review, spatial frequencies, tomography

## Abstract

Diffuse optical imaging probes deep living tissue enabling structural, functional, metabolic, and molecular imaging. Recently, due to the availability of spatial light modulators, wide-field quantitative diffuse optical techniques have been implemented, which benefit greatly from structured light methodologies. Such implementations facilitate the quantification and characterization of depth-resolved optical and physiological properties of thick and deep tissue at fast acquisition speeds. We summarize the current state of work and applications in the three main techniques leveraging structured light: spatial frequency-domain imaging, optical tomography, and single-pixel imaging. The theory, measurement, and analysis of spatial frequency-domain imaging are described. Then, advanced theories, processing, and imaging systems are summarized. Preclinical and clinical applications on physiological measurements for guidance and diagnosis are summarized. General theory and method development of tomographic approaches as well as applications including fluorescence molecular tomography are introduced. Lastly, recent developments of single-pixel imaging methodologies and applications are reviewed.

## 1 Introduction

The ability to probe deep tissues with light for biomedical applications has been recognized since the scientific reports published as early as the late 1800s for monitoring brain hemorrhage^1^ and early 1900s for imaging breast cancer^2^ and performing tissue oximetry.^3^^,^^4^ Since then, diffuse optical imaging techniques have greatly benefited numerous biomedical fields. Mainly, the goal of these optical techniques is to characterize optical properties of tissue using light from the ultraviolet to the infrared spectral region. The specific light–tissue interaction enables monitoring of a vast array of tissue characteristics, including structural, physiological, metabolic, and molecular properties.

The early implementations of diffuse optics were performed using wide-field sources that illuminated large areas of tissues. Such implementations enabled fast and noncontact instruments for ease of use in clinical settings.^5^^,^^6^ However, the collected signal is heavily surface-weighted and contrast in the biomarkers of interests are blurred due to high scattering in biotissues.^7^ Such inherent effects led to an undesirable number of false-positive classifications in breast cancer detection.^8^^,^^9^

In parallel, great strides were made to derive more accurate and efficient light propagation models.^10^^,^^11^ Coupled with the wide spread use of personal computers, such progress led to the development/establishment of quantitative methodologies such as functional near-infrared spectroscopy^12^^,^^13^ and diffuse optical tomography (DOT).^14^ However, such approaches were mainly based on point source and detector configurations. Thus, they were not amenable to imaging large surfaces with high density source–detector configurations. Typically, they relied on a few fibers coupled to the tissue of interest, which led to sampling errors, difficulty in scaling, and high sensitivity to the optode–tissue coupling efficiency.^15^ Due to the recent advent of major developments in spatial light modulators (SLMs), it is now possible to combine both wide-field imaging and quantitative methodologies based on efficient light propagation models to probe large tissue surfaces and retain sensitivity to deep tissues. In this review, we summarize the current progresses in harnessing structured light strategies in diffuse optical imaging. The review covers the three main applications that have shown fast growth over the recent years: spatial frequency-domain imaging, tomography, and single-pixel imaging. Each section will outline the context and founding principles of its technology, followed by advanced methods, current limitations, and applications. The review will be concluded with an outlook of these approaches.

## 2 Spatial Frequency-Domain Imaging

Quantitative sensing of deep tissues or tomography has been largely based on point sources over the last three decades.^16^^–^^18^ Wilson was the first to use full-field structured illumination as a measurement tool, noting that the defocus or blurring of the structure could be used in microscopy for optical sectioning.^19^ In the diffuse regime, Dögnitz and Wagnières^20^ demonstrated that this blurring of structured illumination could be processed to a single value and analyzed as a spatial frequency response that is characteristic of the medium’s optical properties. Wide-field mapping (10  cm×10  cm) of diffuse optical properties was realized by Cuccia et al.^21^ with simple fringe pattern illumination, spatial frequency-domain measurement and analysis, and pixel-by-pixel processing to introduce a method termed spatial frequency-domain imaging (SFDI).

The incorporation of readily available wide-field optical components, the ease of processing in the frequency domain (as opposed to deconvolution in the spatial domain), and the quantitative nature of SFDI has made it a technology worth mentioning—as of this article, Google Scholar shows over 300 citations of the Cuccia et al. 2009 paper and Web of Science shows over 150 publications and over 800 citations for SFDI since 2010.

### 2.1 Introductory Theory, Measurement, and Analysis

#### 2.1.1 Theory

In order to quantitatively map the multiple-scattered photons, i.e., the diffuse reflectance, to tissue optical parameters, a model system must be introduced. The original formulation assumes a homogeneous, linear, time-invariant system. The diffuse reflectance as a function of spatial frequency can be described with a modulation transfer function (MTF) that is characteristic of the sample’s optical properties. From the perspective of signal processing, the highly scattering nature of tissue acts as a low-pass filter. The experimental and theoretical results demonstrate the loss in signal and the shortening of reflected photon pathlengths with increased spatial frequency. This means that the illumination spatial frequency dictates the optical length scale being probed. This mechanism enables depth sensing and quantification techniques.^21^^,^^22^

The first complete measurement and analysis for wide-field mapping of optical properties in the spatial frequency domain was done by Cuccia et al.^21^ and is still the foundation for most SFDI techniques. A brief walkthrough of the conceptual framework will be reiterated here—a thorough description can be found in Ref. 22. It should be noted that the framework is expounded in the diffusion regime for clarity in this context but is not limited therein.

Starting with the time-independent diffusion equation for a homogeneous medium

$$\nabla^{2}\Phi -\mu_{\mathrm{eff}}^{2}\Phi =-3\mu_{tr}q,$$ (1)

gives the relationship between source q, medium properties $\mu_{tr}=(\mu_{a}+\mu_{s}^{\prime })$ (transport coefficient with absorption and reduced scattering coefficients $\mu_{a}$ and $\mu_{s}^{\prime }$, respectively) and $\mu_{\mathrm{eff}}={\left(3\mu_{a}\mu_{tr}\right)}^{1/2}$, and fluence rate Φ. Assuming a semi-infinite geometry, we now introduce a pure spatially modulating source that is periodic and normally incident to the boundary surface

$$q=q_{0}(z)\;\cos(k_{x}x+\varphi ),$$ (2)

which modulates in the x-spatial dimension with spatial frequency $f_{x}=k_{x}/2\pi$ and phase φ and is constant across the y-spatial dimension with arbitrary depth dependence $q_{0}(z)$. Assuming a linear medium, this modulated source will result in a modulated fluence rate with the same frequency and phase

$$\Phi =\Phi_{0}(z)\cos(k_{x}x+\alpha ).$$ (3)

Modulation is allowed for an arbitrary direction and the y-spatial dimension is held constant here for convenience of analysis. These equations are combined to form a one-dimensional (1-D) second-order Helmholtz equation

$$\frac{d^{2}}{dz^{2}}\Phi_{0}(z)-\mu_{\mathrm{eff}}^{\prime 2}\Phi_{0}(z)=-3\mu_{tr}q_{0}(z),$$ (4)

where

$$\mu_{\mathrm{eff}}^{\prime }={\left(\mu_{\mathrm{eff}}^{2}+k_{x}^{2}\right)}^{1/2}\equiv \frac{1}{\delta_{\mathrm{eff}}}.$$ (5)

Here, $\delta_{\mathrm{eff}}^{\prime }$ defines the effective penetration depth, a useful construct for estimating and comparing the depth sensitivity in the spatial frequency domain to other domains. First, note that with $k_{x}=0$ the penetration depth is simply $1/\mu_{\mathrm{eff}}$, as expected from a constant, planar illumination source.^23^ Next, the penetration depth decreases with increasing spatial frequency—a key result that enables depth sensing and tomography (see Secs. 2.2.1 and 3).^21^ Equation (4) is identical to the diffusion equation for continuous planar illumination, and so previous solutions can be utilized with partial-current boundary conditions^24^ to yield the following expression for the diffuse reflectance:

$$R_{d}(k_{x})=\frac{3A\mu_{s}^{\prime }/\mu_{tr}}{\left(\frac{\mu_{\mathrm{eff}}^{\prime }}{\mu_{tr}}+1)(\frac{\mu_{\mathrm{eff}}^{\prime }}{\mu_{tr}}+3A\right)},$$ (6)

where

$$A=\frac{1-R_{\mathrm{eff}}}{2(1+R_{\mathrm{eff}})}$$ (7)

and

$$R_{\mathrm{eff}}\approx 0.0636n+0.668+\frac{0.710}{n}-\frac{1.440}{n^{2}},$$ (8)

where A is a proportionality constant formed from the effective reflection coefficient $R_{\mathrm{eff}}$, obtained by integrating the Fresnel reflection coefficient over all angles of incidence for unpolarized light, and index of refraction ratio n, generalized as a bulk property.

The diffuse reflectance $R_{d}(k_{x})$ is effectively the spatial modulation transfer function of the medium, i.e., the spatial frequency response function of the linear system. As demonstrated in other domains, the diffuse nature of tissue characterizes its response as a low-pass filter in the spatial frequency domain. The diffusion approximation remains valid when $\mu_{s}^{\prime }\gg \mu_{a}$ and when the spatial frequency $f_{x}$ illuminating the sample is much less than the transport coefficient $\mu_{tr}$. For tissue measurements, in practice this means the maximum spatial frequency expected to satisfy the diffusion model is $∼0.33\cdot \mu_{tr}$.^22^ New analytical models have been introduced to characterize the diffuse reflectance beyond the diffusion regime where photons are minimally scattered due to increased absorption or shorter photon pathlengths.^25^^,^^26^

#### 2.1.2 Measurement

Equation (2) depicts a pure sinusoidal source illuminating the sample, but in practice this is impossible—one cannot illuminate with negative intensity—and so a DC offset is necessary to support the AC carrier frequency of source S along the x dimension

$$S=\frac{S_{0}}{2}[1+M_{0}\;\cos(2\pi f_{x}x+\phi )],$$ (9)

where $S_{0}$ is the source intensity and $M_{0}$ is the modulation depth (set to 1 for maximum AC signal). One can see that with a simple 1-D sinusoidal projection, the sample will simultaneously be illuminated with a $S_{0}/2$ DC signal with no modulation and a $S_{0}/2[M_{0}\;\cos(2\pi f_{x}x+\phi )]$ AC wave modulating at $f_{x}$. It is worth noting that this simultaneous AC and DC sampling has been utilized for advanced acquisition methods (see Sec. 2.2.2). For now, consider that the reflected AC intensity $I_{\mathrm{AC}}$ incorporates the tissue’s response in the amplitude envelope $M_{\mathrm{AC}}$ seen here

$$I_{\mathrm{AC}}=M_{\mathrm{AC}}(x,f_{x})\;\cos(2\pi f_{x}x+\phi ).$$ (10)

To retrieve a single measurement $M_{\mathrm{AC}}$ for a given frequency, the most widely used method is the signal processing technique of phase stepping, where three images are collected $I_{1}$, $I_{2}$, $I_{3}$, one at each step of ϕ=[0,120  deg,240  deg]. This procedure is explained for multiple modalities by Mertz.^27^ These images are processed together for demodulation, i.e., to remove the carrier frequency along with the DC signal and to obtain the amplitude envelope at each image pixel $x_{i}$

$$M_{\mathrm{AC}}(x_{i},f_{x})=\frac{2^{1/2}}{3}{\left\{{\left[I_{1}(x_{i})-I_{2}(x_{i})\right]}^{2}+{\left[I_{2}(x_{i})-I_{3}(x_{i})\right]}^{2}+{\left[I_{3}(x_{i})-I_{1}(x_{i})\right]}^{2}\right\}}^{1/2}.$$ (11)

This amplitude envelope $M_{\mathrm{AC}}$ is the product of the initial source intensity $I_{0}$, the optical system’s modulation transfer function ${\mathrm{MTF}}_{\mathrm{sys}}$, and the sample’s response contained in the diffuse reflectance $R_{d}(x_{i},f_{x})$

$$M_{\mathrm{AC}}(x_{i},f_{x})=I_{0}{\;\mathrm{MTF}}_{\mathrm{sys}}\;R_{d}(x_{i},f_{x}).$$ (12)

Because the measurement and analysis is done in the spatial frequency domain, $M_{\mathrm{AC}}$ is in fact the product of the response functions, allowing for a simple ratio against a reference sample of known optical properties to remove the systematic components and retrieve the sample’s diffuse reflectance^22^

$$R_{d}(x_{i},f_{x})=\frac{M_{\mathrm{AC}}(x_{i},f_{x})}{M_{\mathrm{AC},\;\mathrm{ref}}(x_{i},f_{x})}\;R_{d,\mathrm{ref},\mathrm{pred}}(f_{x}).$$ (13)

A light propagation model is used to scale the referenced measurement by $R_{d,\mathrm{ref},\mathrm{pred}}(f_{x})$ and an inverse solver is then used to retrieve optical properties from sample diffuse reflectance measurements. As few as two spatial frequency measurements, one AC and one DC ($f_{x}=0$), can be used to properly generate absorption and reduced scattering maps.^22^

#### 2.1.3 Analysis

**Fig. 1:**
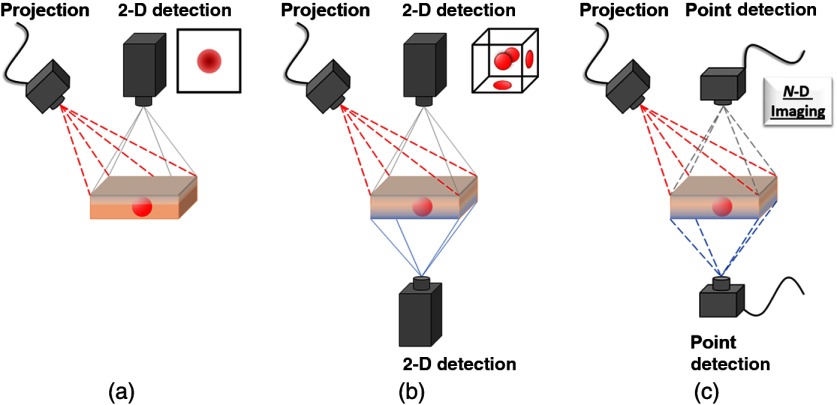
Schematics of typical systems for (a) SFDI, (b) tomography, and (c) single-pixel imaging. Dotted lines indicate structured projection or detection, usually by DMD, and solid lines indicate 2-D camera detection. (a) SFDI offers a simple model, rapid collection, and depth-averaged imaging. (b) Tomography can be in reflection (gray) or transmission (blue) geometry, requires advanced models, processing, and many acquisitions for 3-D reconstructions. (c) Single-pixel detection is highly flexible in its geometry, detection (temporal, frequency, spectral, and/or spatial resolution), for N-D imaging.

The above measurement is analyzed pixel-by-pixel, resulting in a wide-field map of optical properties. Because each pixel is processed independently, technology suited for wide-field use can be readily implemented. System components vary with application, but the most common implementations utilize a digital micromirror device (DMD) for projection and a charged couple device (CCD) or a complementary metal–oxide–semiconductor (CMOS) camera for imaging [see Fig. 1(a)].

To achieve optical property maps of the measured sample, an inverse solver is required for mapping $R_{d}(x_{i},f_{x})$ to $\mu_{a}$ and $\mu_{s}^{\prime }$. While diffusion theory [see Eq. (6)] might be preferred as the forward model and inverse solver due to its speed and ease of use, Monte Carlo models and empirical look-up tables can be precalculated for rapid solutions beyond the diffusion regime.^22^^,^^28^^–^^30^ While simulations in the spatial frequency domain can be done with a complex weighting scheme,^29^ simulations are often done in the spatial domain as a point source and then transformed to the spatial frequency domain, e.g., by radially symmetric 1-D Hankel transform^22^ or by convolution to a line source and a 1-D Fourier transform.^31^

Early analysis methods demonstrated the depth-sensitivity dependence on spatial frequency in diffusive media^21^^,^^32^ and its potential for increased contrast with fluorescent inclusions.^33^ Single wavelength analysis was expanded to multispectral measurements, enabling quantitative chromophore concentration measurements^34^ and mapping of physiological markers with as few as two wavelengths.^35^ To extend SFDI’s clinical relevance, a profile-correction scheme was developed so nonflat samples could be measured.^36^ Acquisition could be relatively fast (on the order of seconds), but this is too slow to avoid certain motion artifacts such as breathing. Therefore, a correction scheme was devised to correct for these artifacts,^37^ though current methods focus instead on rapid acquisition to avoid the issue (see Sec. 2.2.2).^38^^–^^40^

### 2.2 Advanced Techniques

From the fundamentals discussed in the first section, research has expanded the theory, processing, instrumentation, and modeling to develop additional robust techniques that push the limits of quantitative imaging technology.

#### 2.2.1 Advanced theory

Using the diffusion theory result for reflectance [see Eq. (6)], one can derive that with planar illumination ($k_{x}=0$) absorption has its maximum effect on the diffuse reflectance $R_{d}(k_{x})$.^22^ Furthermore, the diffuse reflectance sensitivity to multiple light scattering ($\mu_{s}^{\prime }$) is also maximum in this regime, but increasing spatial frequency, particularly passed the diffuse regime ($k_{x}\gg \mu_{\mathrm{eff}}$), decreases multiple light scattering and increases sensitivity to the scattering phase function. Enabling both diffuse and subdiffuse imaging into a single modality has further demonstrated the utility of structured illumination for imaging biological tissue.

**Fig. 2:**
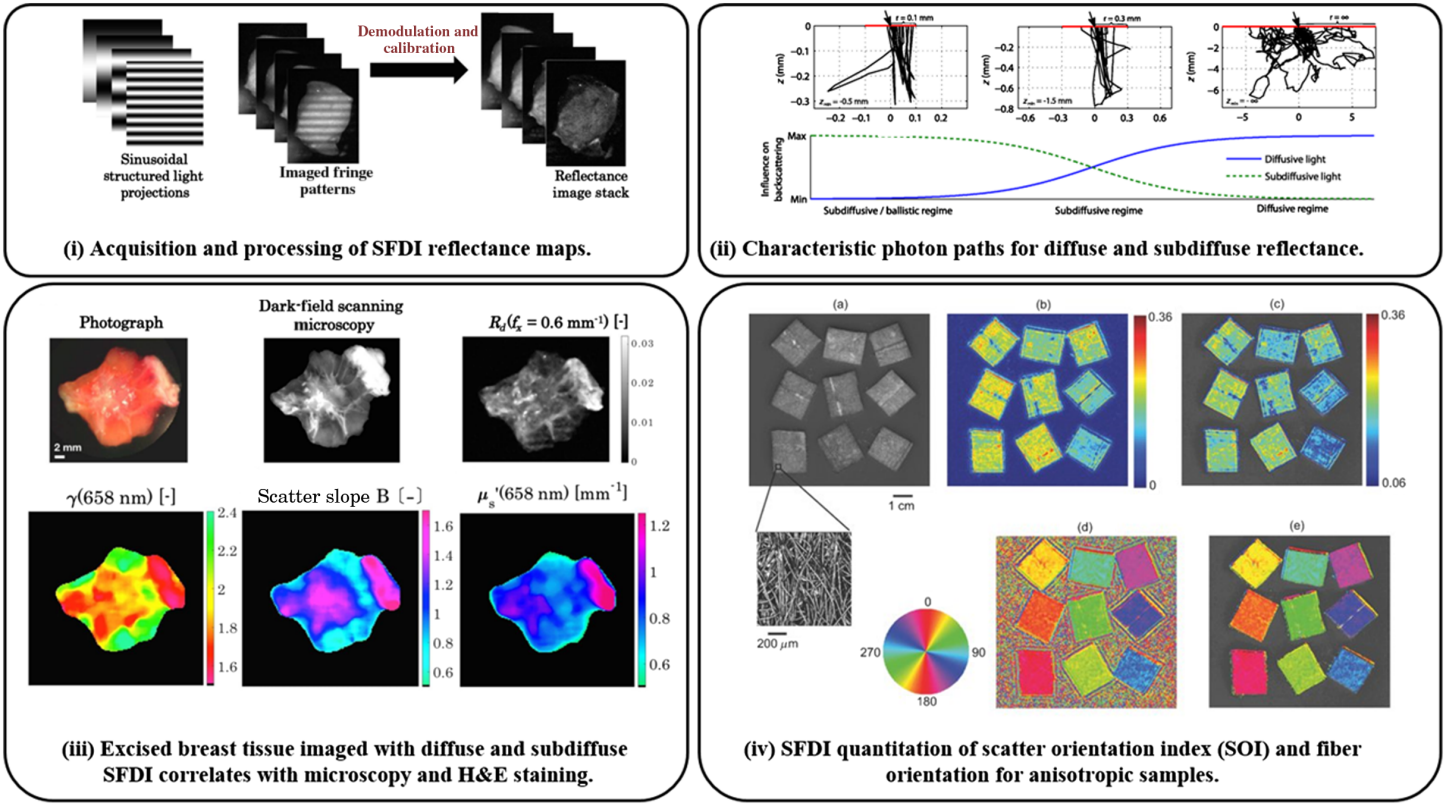
Acquisition, modeling, and application: (i) demonstration of structured illumination patterns, collection, and demodulated reflectance images—adapted from Ref. 45. (ii) Average photon paths get shorter and less diffuse with decreased source–detector separation, similar to increasing spatial frequency—adapted from Ref. 47. (iii) Subdiffuse SFDI demonstrates its ability to measure scattering-related parameters that correlate with histology of excised cancerous breast tissue—adapted from Ref. 45. (iv) SFDI demonstrates sensitivity to the amount of anisotropy (top row) and fiber orientation (bottom row)—adapted from Ref. 48.

Entering the subdiffuse regime [cf. Fig. 2(ii)], studies have shown that with increasing spatial frequency, the reflectance signal becomes increasingly sensitive to the medium’s scattering phase function $P(\theta_{s})$.^26^^,^^41^ Sensitivity beyond the scattering phase function’s first Legendre moment, anisotropy factor $g_{1}=\cos(\theta_{s})$, is often characterized by combining the first two Legendre moments to form scattering parameter $\gamma =\frac{1-g_{2}}{1-g_{1}}$.^42^^,^^43^ With this added phase function sensitivity, studies have demonstrated the characterization of the fractal size distribution of Mie scatterers in phantoms^26^^,^^44^ and McClatchy et al.^45^^,^^46^ even classified tumor grades in *ex vivo* human breast and murine cancer models as compared to histopathology [see Fig. 2(iii)].

However, with the new rapid modeling capabilities of their analytical radiative transport equation (RTE) solution,^25^^,^^49^ Liemert et al.^47^ showed that γ may be prone to errors due to underestimating the number of high-angle scattering events for high spatial frequency reflectance measurements. As a solution, they proposed to weight the higher order Legendre moments of the phase function $P(\theta_{s})$ with a shaping factor c

$$\overset{\infty }{\underset{i=2}{\sum }}{\left(-c\right)}^{i-2}\frac{1-g_{i}}{1-g_{1}}=\gamma -c\delta +c^{2}\varepsilon -c^{3}\zeta +\ldots -\ldots \;$$ (14)

that determines the decreasing weight of the higher order parameters γ, ε, ζ, etc. After extensive modeling for various scattering phase functions and collection geometries, the best average value was c=0.5. This is used to define their scattering parameter σ

$$\sigma =\overset{\infty }{\underset{i=2}{\sum }}{\left(-0.5\right)}^{2}\frac{1-g_{i}}{1-g_{1}}$$ (15)

that outperforms γ in precision.^47^ However, the only testing of σ thus far has been with simulation, and there is extensive literature for comparison of γ measurements in other domains.^42^^,^^43^^,^^50^^–^^52^

Stepping up in length scales from the scattering phase function, wide-field optical imaging techniques generally have poor sensitivity to microscopic scattering structures, such as fiber orientation. However, Konecky et al.^48^ developed an anisotropic diffusion model to simulate and experimentally validate the sensitivity of structured illumination to fiber orientation [cf. Fig. 2(iv)]. By analyzing the amplitude and phase shift of the structured illumination, Konecky et al. deduced the fiber orientations as well as assessed the strength of anisotropy in a scattering orientation index (SOI) defined as

$$\mathrm{SOI}=\max_{\varphi }\{\frac{\left|g(\varphi )-|g(\varphi +\pi /2)|\right|}{\left|g(\varphi )+|g(\varphi +\pi /2)|\right|}\},$$ (16)

where g(φ) is the angular-dependent anisotropy factor of the medium and φ is the angle of the projected spatial frequency.

Broader still, the original modeling of SFDI requires a homogeneous medium, but several studies have developed models and evaluated two-layer systems for a compromise between the gain in depth resolution and the cost of processing and acquisition speed.^53^^–^^55^ These models would go on to support studies evaluating the effects of melanin concentrations in skin on optical measurements,^56^^,^^57^ as well as the layering of intralipid during the phantom experiments.^58^ In addition to correcting for the confounding effects of melanin on chromophore reconstruction using spectroscopy, the depth resolution enables a density measure that is compared to and confirmed with nonfluorescent multiphoton microscopy measurements.^56^

With concern for robustness and quantitation, limitations and sources of error have also been well studied for diffuse structured illumination imaging. A thorough study by Bodenschatz et al.^59^ has developed a list of guidelines to minimize error and to assess the sensitivity of measurement-related parameters. In short, these guidelines state to avoid analyzing the boundary of the illumination field, eschew over-binning camera pixels (though this is minor), limit changes the camera-sample distance or use a correction scheme,^36^^,^^39^^,^^60^ and ensure accurate determination of the projected spatial frequency. Another confounding factor is the popular use of cross-polarization without the proper modeling of polarized photon transport. While cross-polarization is useful to remove specular reflections, this lack of consistency can lead to errors in $\mu_{a}$ of up to 25%.^31^

#### 2.2.2 Advanced processing

The development of SFDI had immediate appeal because of its wide-field, quantitative imaging, but SFDI was mostly utilizing tools and techniques developed for separate spatial, temporal, or frequency domains. With tools and processing built specifically for spatial frequency-domain measurements, new applications are possible and clinical relevance materializes (see Sec. 2.3).

Speed is a major concern for a clinically relevant imaging system. To map the optical properties of a given sample at a single wavelength using SFDI with phase-stepping demodulation for AC signal and DC background subtraction, at least six images need to be processed using Eq. (11) [cf. Fig. 2(i)]. Although this approach is robust, it requires a static sample or a motion-correction algorithm,^37^ though the latest demodulation techniques have minimized the number of required images. A simple approach to cut down the number of acquisitions is to average any three AC images to form the DC image for processing. Nadeau et al.^40^^,^^61^ reduced acquisition requirements down to two images by utilizing a two-dimensional (2-D) Hilbert transform technique. The Hilbert transform technique avoids digital DC filtering by subtracting the DC image from the AC image and seems to keep most of the original image’s resolution, though there is an added complexity of rapidly producing and alternating two patterns and syncing with collection hardware. Nadeau et al.^61^ further advanced rapid acquisition methods by demonstrating the multifrequency capabilities of a square-wave projection. Work by Vervandier and Van de Giessen has pushed acquisition down to a single image using single snapshot of optical properties (SSOP) imaging.^38^^,^^39^^,^^62^^,^^63^ SSOP requires digital separation of DC and AC frequencies that adds complexity to keeping image resolution, though its single image acquisition allows for highly simplified pattern production and image collection. SSOP has been further extended to three-dimensional (3-D) height corrections using 3D-SSOP and has demonstrated real-time imaging of a heartbeat waveform with 50 frames per second.^39^^,^^62^ Another variation that involved single snapshot and multiple frequency modulation also has been proposed, where MTF with respect to spatial frequency provides quantification of absorption and scattering parameters.^64^

Beyond acquisition, further developments have pushed for forward and inverse model solving. Martinelli et al.^65^ developed scaling relationships from the RTE that enable the forward modeling of spatially and temporally resolved reflectance using a single Monte Carlo simulation. Yang and Choi^66^ accelerated Monte Carlo simulations using a graphical processing unit (GPU) specifically with SFDI in mind. Though memory transfer from CPU to GPU is still a limiting factor, a 3400-fold improvement was shown over other GPU-based approaches for multiple diffuse reflectance simulations. Furthermore, recent work by Pera et al.^67^ has demonstrated a method to provide uncertainty estimates of SFDI Monte Carlo solutions for optical properties and chromophore fitting. Based on analytical solutions to the RTE, Liemert et al.^25^^,^^49^^,^^68^ demonstrated several solutions for rapid, highly accurate simulations for various domains in a matter of seconds. This enables quick and extensive parameter testing for model error and measurement sensitivity.^41^^,^^47^ With the aim of enabling real-time processing for clinical use, Angelo et al.^30^ developed rapid look-up table methods that demonstrate a 100-times faster solution over previously published methods.

#### 2.2.3 Advanced systems

The relatively simple and inexpensive instrumentation needed for structured illumination imaging means that expansion and development are inevitable. Demonstrating structured illumination imaging’s potential for broad accessibility, groups have developed low-cost systems made from commercial components that are simplified, portable, and capable of accurate quantitative imaging.^69^^,^^70^ SFDI has also been expanded from the visible and near-infrared (NIR) into the short-wave infrared region by utilizing InGaAs detector arrays.^71^ The spectral density was also increased with work by Singh-Moon et al.^72^ using a line-scan hyperspectral detector. Previous work by Saager et al.^34^^,^^57^ demonstrated spatial frequency-domain spectroscopy point measurements, but 2-D imaging potential lies with hyperspectral point detection using compressed-sensing SFDI.^73^

**Fig. 3:**
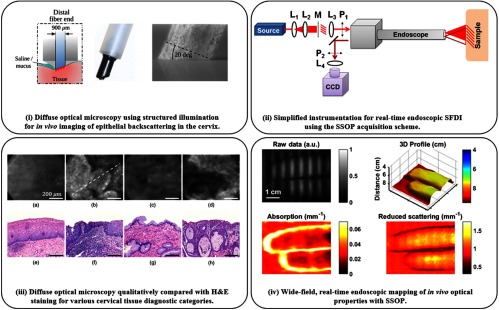
Endoscopic structured illumination imaging: (i) fiber-based probe with structured illumination for diffuse optical microscopy (DOM). (ii) 3D-SSOP enables real-time, quantitative endoscopic wide-field imaging with simplified components: lenses L, mask M, and polarizers P. (iii) (a–d) *In vivo* imaging using DOM of cervical tissue (e–h) with corresponding H&E histology– columns (a) and (c) are benign and columns (b) and (d) are precancerous. (iv) Endoscopic SSOP producing 3-D profile, absorption, and reduced scattering maps from a single raw frame, enabling video-rate imaging *in vivo*. (i) and (iii) are adapted from Ref. 79 and (ii) and (iv) are adapted from Ref. 81.

With an even deeper focus on translation, several groups have developed systems for specific clinical problems. While most are custom in-house systems, the FDA has given its first 510(k) approval to a commercial SFDI system for application in oxygenation imaging for diabetic foot ulcers.^74^^–^^76^ While this brings the standard wide-field system to fruition, efforts by other groups have been made to develop structured illumination endoscopic imaging systems for minimally invasive clinical guidance.^77^^–^^80^ Applying standard three-phase SFDI processing to a fiber-contact endoscope system enables high spatial frequency imaging ($f_{x}=[2.7\text{ to }14.5]\;{\mathrm{mm}}^{-1}$) for cancer detection [cf. Figs. 3(i) and 3(iii)], yet suffers the same reduced overall frame rate as wide-field processing and care must be taken to avoid motion artifacts.^79^ Higher frame rates are achieved with a noncontact, three-dimensional (3-D) corrected endoscopic imaging approach [shown in Figs. 3(ii) and 3(iv)] by utilizing SSOP acquisition and processing, enabling real-time imaging of optical and vascular properties for dynamic samples.^77^^,^^78^ With new technical developments, clinical usage and accessibility of SFDI become practical.

The simplicity of structured illumination imaging lends itself to multimodal measurements with other clinical techniques. Ghassemi et al.^82^ combined out-of-plane Stokes polarimetry with multispectral SFDI to study hypertrophic scars for surface roughness and pathophysiology with a single system. Though each modality has a unique illumination pathway, the shared collection pathway produces inherently coregistered measurements for direct correlation. To better aid surgery and photodynamic therapy (PDT), Rohrbach et al.^83^ performed a feasibility study with SFDI alongside high frequency ultrasound for the combined functional and structural information. Similarly, Lin et al.^84^ obtained multimodal and multiscale images using SFDI, Doppler optical coherence tomography (OCT), and confocal microscopy to study Alzheimer’s disease-dependent changes in a mouse brain model. Furthermore, McClatchy et al.^85^ combined microcomputed tomography (micro-CT) with SFDI to potentially aid in the assessment of tumor margin status in breast conserving surgery. These works open new pathways to clinical efficacy, although much work is needed from the field before multimodal systems come to full form.

### 2.3 Applications

Advanced theory, analysis, and instrumentation have bolstered the understanding of structured illumination imaging while pushing the boundaries of which applications seemed possible.

#### 2.3.1 Photon gating and quantification

The simple projection and collection scheme of structured illumination make it readily adaptable and practical. Slightly different than multimodal systems, some techniques implement the theory, analysis, or processing of structured illumination to directly augment another technique by either gating photons selectively or providing a quantitative foundation.

Because pattern projection is often done with a laser source, laser speckle is a free product that can be utilized for enhanced laser speckle imaging (LSI).^86^^,^^87^ The marriage of wide-field quantitative optical property measurements from SFDI and flow contrast from LSI can be combined to a quantitative, depth-resolved measure of particle flow.^88^ Furthermore, SSOP processing can be implemented to provide real-time quantitative flow imaging.^62^ With the clinic in mind, SFDI/LSI has thoroughly demonstrated potential for real-time burn assessment (see Sec. 2.3.2).^89^^–^^91^

Quantitation has been a long-time goal for fluorescence imaging, so the advent of a wide-field quantitation technique brought a flurry of research to merge the two.^63^^,^^92^^–^^96^ SFDI measurement and analysis of the sample acquire wide-field diffuse optical properties that are then used in a correction model for PDT^92^^,^^94^ and surgical guidance^63^^,^^93^^,^^95^ to account for excitation and/or emission wavelength losses due to absorption and scattering. For SFDI to keep up with fluorescence imaging speeds, SSOP processing can enable real-time optical-property-corrected fluorescence imaging.^63^ While demonstrating substantial improvement over standard fluorescence imaging corrections, SFDI-corrected fluorescence imaging has depth, solvent, and pH conditions that potentially confound the precise quantification of fluorophore concentration, though work has demonstrated that pH quantitation is possible through quantum yield mapping.^96^

**Fig. 4:**
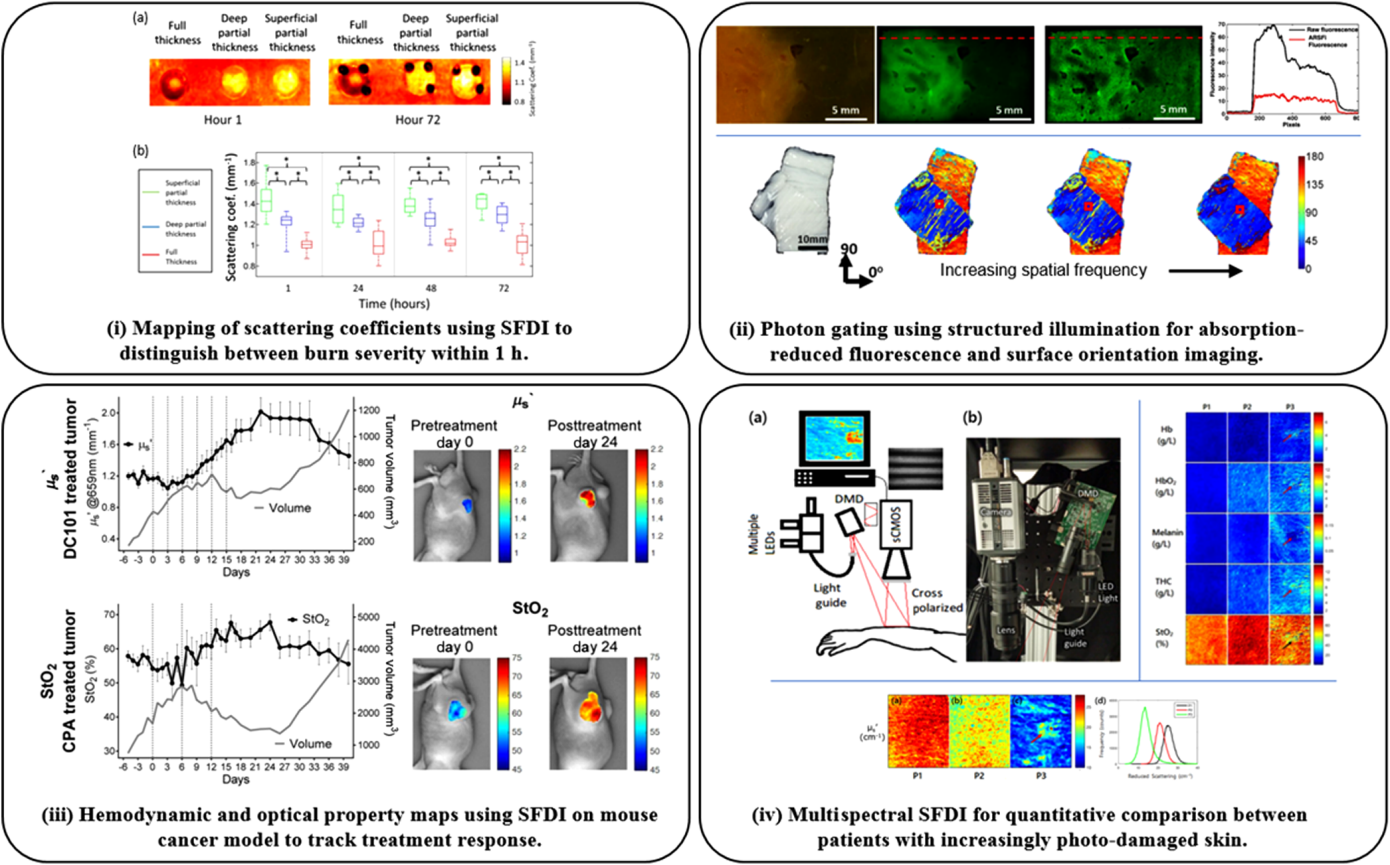
Toward clinical applications using SFDI: (i) SFDI predicts burn severity in a porcine model within 1 h using scattering coefficient imaging—adapted from Ref. 89. (ii) Photon-gating with increased spatial frequency enables absorption-reduced fluorescence imaging (top) and surface fiber orientation imaging (bottom)—adapted from Refs. 101, 102. (iii) Preclinical longitudinal study of tumor growth and chemotherapy response demonstrate feasibility for quantitative tracking of cancer therapies with SFDI—adapted from Ref. 76. (iv) Actinic skin damage assessment using mesoscopic SFDI (top-left) for mapping chromophores (top-right) and the reduced scattering coefficient (bottom) to highlight photodamage in patient P3—adapted from Ref. 116.

Much like polarization, structured illumination can be used as a gating tool to preferentially filter photons by pathlength.^97^ Imaging with increased spatial frequency decreases the average collected photon path length, resulting in a decreased average photon penetration depth^21^^,^^22^^,^^33^ and increased sensitivity to shorter path length interactions.^41^^,^^98^ The spatial pattern can be adjusted during fluorescence imaging to vary the depth and contrast,^33^^,^^99^^,^^100^ or pushed high such that sensitivity to absorption is lost [cf. Fig. 4(ii), top].^101^ This loss of absorption sensitivity comes with added sensitivity to scattering structures and can be utilized for highlighting scattering anisotropies due to fibrous tissue^102^ [cf. Fig. 4(ii), bottom] or skin pathologies.^98^^,^^103^ The benefit of gating with structured light is that it can be dynamically adjusted to suit the depth and contrast needed for the sample’s optical properties. This potentially enables any planar illumination, wide-field imaging technique to capture subdiffuse contrast.

#### 2.3.2 Physiological measurement for guidance and diagnosis

Applying spatial frequency-domain analysis to structured illumination measurements, especially over several wavelengths, provides quantitative ground for further analysis. Some physiological correlations are made directly from optical properties, whereas some techniques perform spectroscopy to recover vascular parameters, and others use fluorescence for guidance and diagnosis.

A first-in-human pilot study for clinical SFDI used during breast reconstructive surgery was performed in 2010.^104^ This feasibility study presented SFDI’s capability for performing oxygenation imaging during a skin flap transplant procedure. Similar models have since been studied in animals to validate the utility of SFDI evaluation of flap profusion and viability.^105^^–^^107^ While skin flap viability is classically assessed by visual inspection, SFDI has shown promise in detecting profusion changes before they are perceptible to the eye by tracking vascular parameters such as total hemoglobin concentration and oxygen saturation.^108^

The functional and structural changes associated with burn wounds, along with their clinically difficult assessment, make the endogenous contrast and noncontact measurement of structured illumination an attractive choice as a monitoring tool. Work has largely focused on the ability of SFDI to predict and distinguish burn wound severity in rat and pig models, demonstrating that vascular parameters can distinguish between partial-superficial and full thickness burns after 1 day^109^ and the structural parameter, i.e., the reduced scattering coefficient, is able to separate all second degree burn severities within 1 h [cf. Fig. 4(i)].^89^^,^^110^ While histopathology is the gold standard for evaluating burn depth, it is often avoided due to its invasiveness. However, a recent study successfully correlated the invasive histopathology of a porcine burn model with the noninvasive combined sensitivity of SFDI and LSI to corroborate their potential for clinical use.^90^ Similarly, other combinations for multimodal systems, e.g., polarimetry with spectral SFDI^82^ and laser Doppler imaging with SFDI,^111^ have been used to investigate burn wound infections and scar formation with noncontact endogenous imaging.

Brain imaging with structured illumination has shown potential for several applications. Alzheimer’s disease in mouse models can be detected with the functional imaging of SFDI,^84^^,^^112^ and the corresponding neuronal cell death correlates to its scattering parameter.^113^ A major push for quantitative-fluorescence imaging is for tumor targeting, particularly for precious tissue such as the brain. As demonstrated by Konecky et al.^114^ and Sibai et al.,^115^ SFDI can be used to quantify the fluorophore concentrations in a wide-field mapping for glioma resection. In addition, brain imaging with hyperspectral SFDI was correlated with point-based optical pharmacokinetics measurements to successfully trace drug delivery concentrations to glioma tumor sites.^72^

The spatial heterogeneity of cancer growth and the associated high rates of secondary excisions make the spatial mapping and quantitative sensing of structured illumination a potentially powerful clinical aid. In a broad view, SFDI has been used in preclinical models to monitor the efficacy of cancer therapies to aid diffuse optical methods in the clinic [cf. Fig. 4(iii)].^76^ Aimed at direct clinical intervention, several studies have demonstrated the potential of structured illumination for specific tissue-type diagnosis in *ex vivo* tissue, such as breast tissue (diffuse^74^^,^^117^ and subdiffuse^45^) and ovarian tissue.^118^ Both the functional and structural properties measured with structured illumination help quantify tissue viability.^46^^,^^74^ Furthermore, the pilot study for a nonmelanoma skin cancer clinical trial with SFDI optical and vascular parameters presented a clear separation of healthy tissue and each lesion stage of precancerous actinic keratosis as seen in Fig. 4(iv).^116^

## 3 Tomography

### 3.1 Introduction and General Theory

Fast and quantitative mapping of optical contrasts over large surfaces benefits numerous biomedical applications, but the fundamentals of light–matter interaction typically limit the use of single-projection imaging techniques to the superficial sensing of tissues without the ability to retrieve depth information and 3-D shapes. When deeply embedded contrasts are targeted and 3-D volumetric imaging is required, more sophisticated methods that involve an inverse optical problem are employed and are broadly labeled optical tomography. Optical tomography can be performed at multiple length scales ranging from optical projection tomography, which focuses on millimeter-scale transparent specimens,^119^ to mesoscopic fluorescence molecular tomography (FMT) and macroscopic DOT, both of which harness scattering photons to retrieve the biodistribution of biomarkers of interest a few millimeters deep or over centimeter scales, respectively. Herein, we will focus on macroscopic DOT and the readers interested in the mesoscopic regime can refer to Ref. 120.

Macroscopic optical tomography principles were developed in the late 1980s and early 1990s with focus on retrieving absorptive inhomogeneities deep within a sample. Over the years, the technique(s) has been improved to image multiple endogenous biomarkers and termed DOT. In addition, the principles of DOT have been adapted to image fluorescence signals and for fluorescence applications, termed FMT.^121^^–^^125^ In both cases, traditional macroscopic tomography systems were designed around multiple point sources or detectors that were raster-scanned or consisted of large fiber bundles, and some systems contained a stage for rotation of the sample or the source and detector planes.^126^ Eventually, many researchers adopted CCD cameras as the detection scheme for parallel acquisition and illumination. DOT can be performed efficiently using continuous wave (CW)^127^^,^^128^ systems with the advantage of robustness and ease of implementation. More challenging are frequency-domain^126^^,^^129^^,^^130^ and time-domain instrumental implementations.^125^^,^^131^^,^^132^ However, these implementations enable the measurement of time-dependent data sets such as modulated amplitude and phase,^14^^,^^133^ time gates,^134^ or transformed data^135^ with the benefit of higher information content for more accurate optical tomographic reconstructions.

To perform optical tomography, the volume to be imaged is discretized in elements of volume and the inverse problem aims at retrieving the value of the biomarker of interest in each of these volume elements (or x below). To do so, a photon propagation model of the light fluence is generated in order to calculate the contribution, or “weight,” of each volume element in the sample to the overall measurements. Then, a classical inverse problem is formulated, i.e., Ax=b, where A is the Jacobian (or sensitivity) matrix generated from the forward model and b is the measured data, and then solved to determine the values of x over the whole volume. The main principles for reconstruction have not altered significantly over time, but due to developments in computational power, there have been great improvements in the efficiency and accuracy of reconstruction algorithms. For simple geometries, researchers have generated the sensitivity matrix analytically, but higher-complexity samples require numerical solutions computed via the finite element method^136^ or Monte Carlo methods.^126^^,^^137^^,^^138^ Computationally efficient Monte Carlo platform for structured light applications can be found at http://mcx.space/.^139^ Solving the inverse problem is not trivial and many different methods have been developed,^124^^,^^125^^,^^128^^,^^137^ but a description of these methods is beyond the scope of this review. Overall, these traditional DOT/FMT methods enable reconstruction of properties (position, absorption/scattering coefficient, and fluorescence yield) *in vitro*^122^^,^^132^ as well as *in vivo* in small animals^140^ with high sensitivity and relatively high resolution.

Despite the widespread use of DOT/FMT with point sources and detectors, there are many limitations. On the acquisition side, raster-scanning point illumination and detection methods can lead to overly long acquisition times due to the necessity to acquire dense spatial data sets. The use of point sources also limits the power density that can illuminate the sample at each point, to avoid photobleaching in fluorescent samples or damage to tissue for *in vivo* samples. Moreover, such methodologies are not amenable to scaling to large volumes without loss of volume sampling. On the reconstruction side, since there are many sampled source–detector pairs, the measured data sets become very large as does the sensitivity matrix generated from the forward model used in solving the inverse problem. This results in high computational burden, which limits the postprocessing method despite improvements in hardware and software.

### 3.2 Method Development

Early techniques such as phased array^141^ were proposed to mitigate the above-mentioned issues and boost the sensitivity^142^ and resolution of DOT.^143^ However, these early concepts of structured excitation of tissue are based on photon density wave interference that can be challenging to generate and control. It is nowadays relatively straightforward to generate spatially modulated wide-field illumination and detection methodologies using DMDs to harness the potential of structured light strategies. Hence, researchers have increasingly begun to investigate wide-field detectors and structured illumination to efficiently perform DOT.

As a more general tomographic approach, utilizing structured light of any arbitrary shape can be utilized for tomographic imaging. Some of the main expected benefits of structured light strategies are reduced number of required measurements due to the inherent wide-field nature of structured light and the potential for harnessing compressive sensing methodologies. Indeed, CS methodologies have been developed over the last decade to help reduce the size of digitally large data sets during the acquisition step. Since CS can take advantage of the sparsity of the image sample plane when illuminated with a determined patterned basis, the desired image can be recovered with far fewer measurements. Conveniently, structured light approaches enable one to efficiently implement this by selecting a sparse illumination basis. Therefore, CS allows for data compression during the acquisition step, which is beneficial for acquisition time reduction.^144^ In addition, the samples can be illuminated with a decreased overall power density due to the large illumination areas leading to relaxation of safety and photobleaching issues. Since one can change the shape and intensity at or close to region of interest where the signal is relatively low due to high absorption (e.g., liver in small animal imaging), an increased SNR can be achieved in those areas due to efficient illumination strategy related to the target site.

**Fig. 5:**
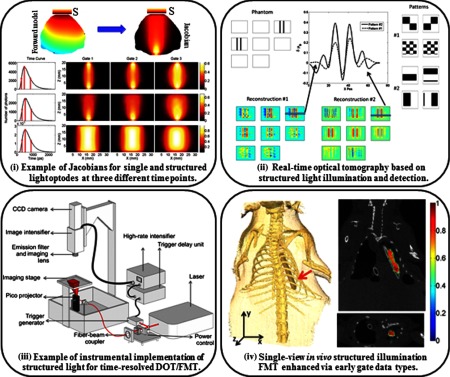
Light tomographic implementations: (i) simulated detector readings for the central source-pattern and associated Jacobians for the three gates selected: point source–point detector (top row), patterned illumination–point detector (middle row), and patterned illumination–patterned detection (bottom row adapted from Ref. 145). (ii) Simulated phantom reconstructions based on two types of patterns: phantom used for the simulations (top left), two types of patterns for illumination and detection (top right), reconstructions for each pattern type (bottom), cross sections and quantitative values of the differential absorption across a z-slice. The volume for the reconstruction is divided in 16z-slices, each having 1.25-mm thickness; even z-slices are shown (top middle adapted from Ref. 146). (iii) Wide-field fluorescence lifetime imaging system for *ex vivo* and *in vivo* imaging. The schematics of the time-domain fluorescence lifetime imaging system based on a gated ICCD detection are shown (from Ref. 147). (iv) 3-D volume from the CT scan showing the position of the tube in the chest cavity (left). Coronal slice of the reconstructed volume at z=6:5  mm. (Bottom right) Transverse slice of the volume at y=21:5  mm (top right adapted from Ref. 148).

These implementations come at the cost of slightly increased computational complexity as modeling of spatially complex sources is required in the forward model simulation for generation of the sensitivity matrix [cf. Fig. 5(i)]. However, flexible, accurate, and universal computational Monte Carlo tools are now widely available to generate such sensitivity matrices both in the case of structured illumination as well as detection.^139^

The first investigations of the potential of structured illumination in DOT were performed using simulations. Lukic et al.^149^ proposed the use of structured illumination for tomography in the frequency domain, and they demonstrated *in silico* that reconstructions performed with structured illumination patterns provided comparable resolution to those generated using point-source illumination. In addition, they showed that the amount of data acquired using structured light was decreased by a factor of five compared to a previously tested point-source illumination system. Similarly, Joshi et al.^150^ proposed the use of structured-light tomography in the time-domain *in silico* but in the reflectance geometry. The researchers compared a scanned line pattern, scanned Gaussian spot patterns, patterns such as a cross, and a series of equally spaced lines that can be generated with diffractive elements, and point sources on simulated phantoms with fluorescence inclusions. They showed that structured illumination patterns outperform point-source illumination in terms of resolution and location accuracy for multiple fluorescent inclusions close to the surface. Each pattern type provided a slightly different reconstruction result, which suggested that the patterns need to be optimized according to the sample of interest. In this regard, Dutta et al.^151^ developed an optimization framework for generating optimal spatial illumination patterns for CW FMT based on an approach that seeks to improve the condition number of the Fisher information matrix. However, this methodology is computationally expensive and requires prior knowledge of sample surface topography and tissue optical properties, which makes it difficult to implement practically *in vivo*.

These simulation studies paved the way for current implementations of structured-light tomography. Spatial frequency-domain tomography utilizing sinusoidal spatial illumination was first implemented in reflectance geometry by Konecky et al.^32^ by solving the heterogeneous diffusion equation with a linear perturbative approximation in Fourier space. The authors demonstrated the feasibility of fast tomographic reconstruction of absorption contrast due to the analytical expressions based on Green’s functions and the Rytov perturbation model. The work was later extended for 3-D reconstructions of fluorescence contrast with the clinical application of surgical guidance of glioma resection (cf. Fig. 5).^114^ For full volume tomography beyond a couple of millimeters, Bélanger et al.^146^ implemented structured-light-based strategies in transmission and compared two initial sets of 36 wide-field patterns, namely checkerboard and low spatial frequency bar patterns that illuminated half of the sample area in each pattern, in a system with structured illumination and wide-field detection. These patterns were tested in centimeters-thick simulated phantoms as well as liquid phantoms containing graphite rods with 100% absorption. Their results showed that it was possible to reconstruct inclusions with high resolution and accurate locations with a smaller number of measurements compared to the data sets collected with traditional tomography systems, leading to near-real-time acquisition speeds. In addition, it was determined that the set of bar patterns provided better reconstruction of absorption contrast and showed higher robustness to noise compared to the checkerboard pattern set [cf. Fig. 5(ii)]. This work was closely followed by the implementation of structured illumination for time-resolved preclinical studies.^147^ The schematic of the system developed by Venugopal et al. is provided in Fig. 5(iii). Using the same bar patterns as Bélanger et al., Chen et al.^145^ demonstrated *in vitro* that these bar patterns provided accurate reconstruction of absorptive inhomogeneities. Also, leveraging the time-resolved data sets, Venugopal et al.^147^ demonstrated the ability to quantitatively reconstruct both absorption and scattering contrast with minimal cross talk. Lastly, Venugopal et al.^152^ were the first to demonstrate the utility of the technique in imaging fluorescence signals in small animals at fast acquisition speeds. The combination of Monte Carlo-based forward simulations^153^ that enable harnessing of the early photons for improved resolution and late photons for quantification^134^ led to accurate reconstructions even in the case of a single-projection system.^154^ An example of time-resolved enhanced FMT reconstruction is provided in Fig. 5(iv). Ducros et al.^128^ also compared these data types in the reflectance and transmission geometries, and they came to similar conclusions regarding the use of time-resolved data.

The bar patterns used in these pioneering studies were selected based on the work of Bélanger et al.^146^ but also due to the experimental ease of implementation for complex geometries such as in small animal imaging. Still, numerous spatial bases can be considered and implemented such as typically done in the field of compressive sensing. To date, a few studies have investigated the use of well-known bases for structured-light tomography with the goal of improved compression of the measurement space, such as measurements obtained with wavelet-based patterns^155^ and piecewise-constant functions.^156^ Moreover, theoretical CS bases typically contain negative components that cannot be directly projected onto the sample. Following well-established imaging protocols in CS-based 2-D imaging, Ducros et al.^157^^,^^158^ proposed a virtual source pattern method in which the patterns, which can be negative or have a complex component, are transformed into a projectable pattern with positive intensities using a transformation matrix. This transformation matrix is then applied to the measured data as well as the calculated sensitivity matrix for reconstruction of the inclusion. Methods have also been developed to decrease the overall computation time of tomography, such as the initial projection of sinusoidal fringe patterns to construct *a priori* surface profiles for generation of the forward model.^159^^–^^161^ In addition, structured light strategies are well-suited to benefit from CS-based preconditioning of the illumination and detection fields for improved performance.^162^ Beyond the potential of using optimal illumination patterns based on theoretical considerations, structured light methodologies are also amenable to implementing strategies to improve the SNR of the measured data set. For instance, Zhao et al.^163^^,^^164^ demonstrated that by iteratively optimizing the local illumination field, sensitive topographic imaging in preclinical models was achievable. Venugopal and Intes^165^ proposed a similar concept for 3-D imaging and demonstrated that the number of useful data sets could be increased by twofold using this methodology. Such an approach is poised to enable the collection of optimal data sets without requiring any prior knowledge. These developments have laid the foundation for wide dissemination of structured-light tomography. As the technique is adopted by an ever-increasing number of research groups in the world, more applications are benefiting from such improvements.

### 3.3 Applications and Further Developments

#### 3.3.1 In vivo fluorescence molecular tomography

FMT has become popular over the last two decades due to improvements in and availability of NIR fluorescent dyes, biomarkers, and reporter genes that can be used *in vivo*. The three major benefits of FMT for *in vivo* molecular studies are its high sensitivity that can rival nuclear imaging, its ability to simultaneously image multiple biomarkers via spectral and lifetime encoding, and the unique wealth of information that can be derived from lifetime sensing such as microenvironment parameters or protein–protein interactions. To date, FMT in preclinical settings has been the main application of structured-light tomography.

**Fig. 6:**
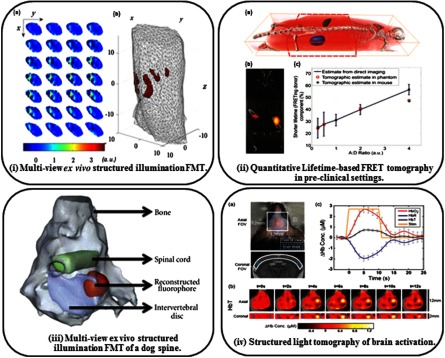
Applications of tomographic reconstruction using structured illumination: (i) of fluorescent tubes implanted within a freshly euthanized mouse. The data shown were taken by moving low spatial frequency quantized bar pattern illumination and wide-field detection. The data shown were collected with virtual source Haar wavelet patterns, (a) the raw fluorescence data for multiple views and (b) the reconstructed inclusions within the 3-D mesh.^166^ (ii) Tomographic quantification of FRET. Reconstruction of the fluorescence quantum yield is shown in (a) overlay onto a coregistered microCT model in 3-D and (b) in a cross-sectional slice. (c) The quantification of FRET through use of the FRETing donor fraction of the two FRET inclusions is similar to the values extracted from tomographic imaging of the mouse, a separate phantom with four FRET inclusions, and direct planar imaging.^148^ (iii) Reconstructions of ICG distribution in an *ex vivo* dog spine using multiview time-resolved structured light illumination and mesh-based Monte Carlo with nonconcurrent MRI.^167^ (iv) Structured light tomography of brain activation in a live mouse. (a) The imaging area is shown in axial and coronal slices. The paw of the mouse was stimulated at the beginning of the imaging session, and (b) the distribution of total hemoglobin HbT within the brain was seen to change according to activation of various areas of the brain. (c) The time-course response in terms of total hemoglobin, oxygenated hemoglobin (${\mathrm{HbO}}_{2}$), and deoxygenated hemoglobin (HbR).^171^

Venugopal et al.^152^ were first to demonstrate *in vivo* accurate reconstructions of fluorescent inclusions inserted into a freshly euthanized mouse. The performance of this single-projection structured light approach was cross-validated with nonconcurrent CT scans. Of note is that no *a priori* information was included in the optical reconstruction. As shown in Fig. 5(iv) in an overlay with coregistered microCT data, the inclusion was reconstructed with high accuracy due to the early gate data type and sparsity-enhancing solvers.^154^ Similarly, Ducros et al.^166^ later demonstrated the use of experimentally adapted virtual source Haar wavelet patterns for FMT of a fluorescent inclusion implanted within a euthanized mouse in a system with a rotating stage. First, the mouse was imaged at multiple rotation angles using a wide-field pattern for generation of adapted patterns as well as the model for forward calculations. These patterns were then used for imaging of the mouse at eight rotation angles to enable reconstruction of the fluorescence inclusion. As shown in Fig. 6(i), the inclusions were accurately reconstructed in terms of the locations and dimensions, but the authors note that there are still artifacts within the reconstruction that can be improved with the use of *a priori* information. Still in both the cases of Venugopal et al. and Ducros et al., the 3-D data acquisition was performed at fast acquisition speeds and enabled whole-body 3-D imaging.

Demonstration of the ability to image an *ex-vivo* preclinical model was also demonstrated by Pimpalkhare et al.^167^ The specimen employed was a dog spine model injected with a small dose of indocyanine green (ICG), a common NIR fluorophore. Multiview time-resolved-structured light tomography was performed and accurate distribution of the fluorophore as retrieved in this complex sample was validated by microMRI cross-validation [cf. Fig. 6(iii)]. Interestingly, the authors noted that improvements in resolution were more pronounced when using multiple views compared to leveraging of early gate data sets. However, implementation of multiview systems for preclinical studies is cumbersome and typically done by rotating the sample in a vertical position. This is not a natural physiological posture for animals and leads to challenges when performing nonconcurrent imaging with other common modalities that are designed around a prone position.

There are currently still more efforts to improve efficiency and accuracy of structured light FMT. For instance, noncontact and nonrestrained preclinical imaging leads to the projection of theoretical patterns on complex boundary conditions. SLMs are flexible enough to enable the scaling of patterns in real-time to encompass the boundaries of the animal. Then, to ensure reconstruction accuracy, *a priori* information about the projected patterns should be included in the forward model via a complex modeling scheme such as a mesh-based method.^138^^,^^139^ Current forward solvers such as GPU-enhanced finite element Monte Carlo are particularly adept for such complex modeling.^138^ Moreover, in combination with computationally efficient formulations,^168^ whole Jacobians, even in the time-resolved cases, can be computed in a matter of minutes on a personal computer. Combined with mesh optimization techniques,^169^ they offer the unique attributes of accuracy for all kinds of diffuse regimes, fast computational times, and improved resolution via mesh refinement.

#### 3.3.2 Lifetime-based tomography

Beyond establishing the potential of structured-light tomography for retrieving the biodistribution of fluorescent markers, Venugopal et al.^148^ demonstrated the utility of the methodology to image lifetime-based contrast. Fluorescence lifetime can be defined as the intrinsic property of a fluorophore of reaching an excited state that will lead to the emission of photons, to then return to its initial ground state.^170^ Since it is an intrinsic property, it is independent of concentration, tissue depth, or photobleaching, being only affected by extrinsic factors such as temperature or quenching.^170^ More precisely, the authors demonstrated the ability of time-resolved structured-light tomography to quantify Förster resonance energy transfer (FRET) occurrence via sensing of the donor lifetime.

In brief, FRET is a phenomenon that occurs when two molecules that have high spectral overlap, denoted the donor and acceptor, are within 2 to 10 nm apart. At this distance, the donor transfers energy to the acceptor, which causes the intensity and lifetime of the donor to decrease while those of the acceptor increase. By measuring the changes in intensity or lifetime of the donor, FRET can be used as a nanoscale proximity assay *in vivo*. Lifetime-based FRET has already been established as a useful tool for monitoring ligand–receptor engagement *in vivo* in wide-field planar imaging of cancerous tissue.^172^^–^^174^ Tomography of FRET was validated by Venugopal et al.^148^^,^^175^ using an NIR FRET pair, AF700-AF750. For the *in vivo* study, the moving low spatial frequency bar patterns optimized with the adaptive optimization method^165^ were utilized on a freshly euthanized mouse with two inclusions of different FRET ratios. As shown in Fig. 6(ii), the locations and fluorescence yields of the two inclusions were accurately reconstructed. In addition, it is shown that the tomographic reconstruction of the donor population that interacts with the acceptor (denoted the FRETing donor and characterized by a quenched lifetime) is consistent with the two inclusions from tomographic reconstruction of FRET in a phantom experiment as well as from direct nontomographic imaging. The quantification of the FRET donor via tomographic imaging was reported to be within 5% of the value quantified on the same system but without any surrounding scattering tissue.

#### 3.3.3 Functional imaging

Structured-light tomography is also well-suited to image endogenous markers. The improvement in acquisition speed positions the technology favorably for any applications targeting hemodynamics. This is a nascent application with great promise. Recently, Reisman et al.,^171^ following the pioneering developments highlighted above, implemented structured-light tomography for functional brain imaging of the intact mouse brain (through the scalp and skull). The researchers collected the data by projecting sinusoidal patterns of different spatial frequencies, phases, and orientations. The sensitivity matrix was generated using a finite element mesh and the diffusion equation, and the local absorptive perturbations associated with modulation of the hemodynamics were reconstructed. As shown in Fig. 6(iv), changes in blood flow were realized upon stimulation of a limb, which was characteristic of activation of the specified brain region. This method is especially powerful since it allows for monitoring of different hemodynamic properties noninvasively in a live mouse. Imaging of brain activation has typically been performed using NIR spectroscopy, but incorporating structured-light tomography shows improved recovery of focal brain functional activation. Such implementations may find numerous other applications in functional imaging such as optical mammography^176^ or monitoring of perivascular diseases.

## 4 Single-Pixel Imaging

### 4.1 Introduction

Another rising application of structured light strategies in diffuse optics is the implementation of single-pixel camera methodologies. The combination of SLMs with compressed-sensing approaches enables the development of imaging systems based on one detector that nevertheless can provide 2-D imaging capabilities without any moving parts. Even though CCD and CMOS light sensors have improved throughout the years, their application for spectral bands beyond the visible regime is still limited since they mostly operate in the visible range and producing them for the NIR and infrared range is complex and expensive.^177^ Conversely, single-pixel systems can leverage detectors sensitive to these spectral bands and provide 2-D imaging capabilities at a reduced cost. The standard setup of a single-pixel system is composed of a DMD, which is used to project patterns onto the sample plane, and a single-pixel detector that collects the sample emissions after illumination. This classical setup has been often modified into more complex systems with enhanced detectors or illumination schemes. These systems can also adopt different scanning methodologies that can yield better intensity reconstructions or improved acquisition times.^177^

As described by Welsh et al.,^178^ ghost imaging, the precursor of single-pixel imaging, initially consisted of two detectors with low and high resolutions, respectively. The first one collected the light scattered from the sample and the second one detected the unscattered illumination scheme. The incident light could be generated by pseudothermal light sources^179^ that produced speckle-like illumination. A beam splitter would duplicate the illumination arrangement for it to be sampled by the high-resolution detector. The data collected from both detectors could later be used to retrieve the sample’s image. The use of this detector–beam splitter complex was later unnecessary once SLMs were introduced. An SLM would computationally produce structured illumination where parameters such as phase and intensity were regulated. Therefore, the use of known illumination fields removed the need of a high-resolution detector. Hence, the setup was simplified to one detector and an SLM for illumination, which is the basic scheme of a single-pixel detection system. Combining the single-pixel detection scheme with compressive sensing,^177^ which takes advantage of the sparseness of the acquired data, reduces the amount of required data to reconstruct the sample’s image.

The main advantages of single-pixel implementation are twofold. First, compared to other imaging schemes that depend on expensive pixelated detectors that respond only to visible wavelengths of light, single-pixel imaging uses a single detector which can, when selected judiciously, sense desired wavelengths outside the visible range.^180^ Numerous applications in the biomedical field can benefit from cheaper and better sensitivity in these spectral bands,^7^^,^^181^ such as infrared imaging,^182^ hyperspectral imaging,^183^ or 3-D imaging.^184^^,^^185^ Second, single-pixel detectors can work better under poor light conditions.^181^ This is mainly associated with more sensitive detectors but also due to the spatial integration of the optical signals collected at each illumination pattern. It is also of note that single-pixel systems can be extended to the time-domain for efficient wide-field lifetime imaging applications. The basic setup can be enhanced by adding a time-correlated single-photon-counting unit (TCSPC), resulting in a time-correlated single-pixel system.^183^ Time-correlated systems can be later analyzed to investigate properties such as fluorescence lifetime^186^ or FRET.^187^ Unfortunately, in the classical single-pixel detection configuration, a high number of patterns are needed to yield a good image reconstruction, which also correlates to higher exposure times for the sample. Moreover, the quality of the reconstructed images is lower than the one obtained using a common pixelated detector. These disadvantages have been overcome through the years by fusing the technique with theories such as compressive sensing.

### 4.2 Methods

**Fig. 7:**
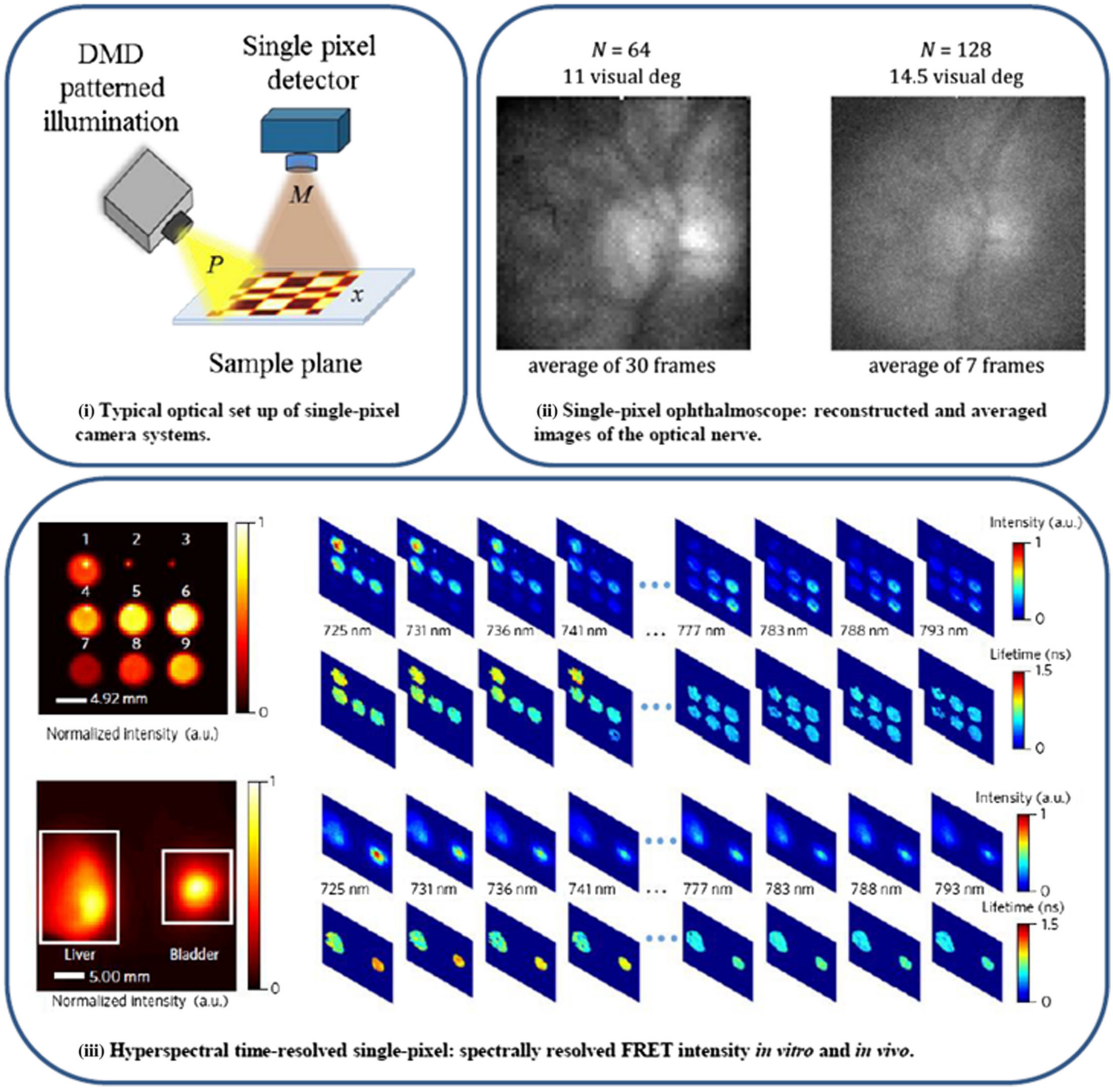
(i) Simplified optical setup of the single-pixel camera using a DMD. The image is noted x, P is a DMD pattern, and M is the corresponding measurement.^181^ (ii) Living human eye imaging using a single-pixel camera ophthalmoscope.^188^ (iii) Spectrally resolved FRET intensity imaging *in vitro* and *in vivo*.^187^

A single-pixel system typically follows the design proposed by Duarte et al.^177^ with slight modifications based on the application at hand. Overall, a single-pixel camera is composed of a single-pixel detector, an SLM for pattern illumination, and relay optics as shown in Fig. 7(i) by Rousset et al.^181^ The SLM typically is a DMD, which gives the advantage of producing both binary and grayscale structured illumination. The angle of the DMD micromirrors can be digitally manipulated to filter a determined amount of light for that specific area. All the mirrors in conjunction can then produce grayscale patterns with up to 10-bit resolution.^181^ The characteristics of the basic components are dependent on the application of the system. The detectors can vary from “bucket” detectors^189^ that have no spatial resolution and directly detect all the scattered photons, e.g., single photodiodes and photomultiplier tubes, to multichannel detectors^187^ that detect dispersed light in multiple wavelength channels. The relay optical elements vary depending on the type of detector and illumination scheme, for example, they can adopt lensless configurations^190^^,^^191^ or use elements such as physical masks.^192^ Optical setups can also vary from single-pixel microscopy systems^193^^,^^194^ to macroscopic approaches.^188^^,^^195^

#### 4.2.1 Scanning methodologies

Beyond the consideration of the best optical elements and detectors for the application at hand, one must also select a proper scanning methodology. Indeed, as mentioned by Duarte et al.^177^ a single-pixel camera can operate under different acquisition methodologies such as the basic principle of acquiring N measurements to reconstruct an N-resolution image or under multiplexing strategies such as compressive sensing. These scanning methodologies must take into consideration the signal-to-noise ratio during the acquisition process, which can be diminished by Poisson noise or the general instrument response. If an N-pixel image is to be reconstructed through single-pixel measurements, it can be typically done by using a raster scan, a pixel array, or a basis scan. In the latter case, basis scanning is constructed using a single sensor that will detect N measurements acquired one after the other with patterned illumination, where the patterned light will target diverse arrangements of the N pixels on the image plane.^177^ Multiple types of illumination basis can be employed, the most commonly used include Hadamard, speckle, Fourier, and orthogonal, or biorthogonal wavelets such as Haar, LeGall, and Daubechies. Furthermore, basis scanning can take advantage of the image plane sparseness to only require part of the full basis patterns to reconstruct the image plane through compressive sensing algorithms.^177^^,^^196^^,^^197^ Basis scanning performs better than raster scanning by reconstructing similar intensity images at much lower capture times.^177^ Therefore, compressive sensing reduces the number of patterns per total acquisition, consequently reducing the exposure time. This is key in cases where the sample is sensitive to photodamage within the expected complete acquisition time.

#### 4.2.2 Image recovery approaches

For single-pixel camera systems, the 2-D image sought is not obtained via direct imaging but via an inverse problem. Indeed, the data acquired during basis scanning with a single-pixel camera is the inner product of the illumination patterns and the sample, therefore retrieving the sample’s intensity profile through processing algorithms is necessary. This inverse problem is rather simple and can be expressed as

M=ΔtPx, (17)

where M is the single-pixel measurement over a Δt acquisition period, P denotes the set of illumination patterns used for acquisition, and x represents the image of the sample plane.^181^ Compressive sensing assumes that the sample can be sparsely represented by a basis α. Therefore, x=αs, where s represents the sparse image plane. Note that the illumination basis has to satisfy the “restricted isometry property.”^198^ Solving a minimization-optimization problem for that particular transform-domain can then retrieve the sample’s image.^181^

#### 4.2.3 Pattern selection

One area of focus for the field of single-pixel imaging is the selection of optimal basis for fast and accurate 2-D image reconstructions. Common bases include Hadamard, speckle, Fourier, and wavelet-based patterns. Streeter et al.^199^ explained that Hadamard multiplexing is known to improve the SNR in the acquired data by decreasing additive noise. Two main types of Hadamard matrices are commonly used, the H-matrix which is composed of ones (1’s) and minus ones (−1’s) and the S-matrix which is formed by zeros (0’s) and ones (1’s). Since the S-matrix is easier to implement in SLMs, it is more commonly employed for structured illumination than the H-matrix, even though the latter should provide an even better SNR boost. Although the additive noise is decreased, Hadamard patterns are affected by Poisson noise generated by photons, which can decrease the SNR of the measurements.^199^^–^^201^

As described by Guo et al.,^191^ speckle patterns can also be implemented in single-pixel systems, where the resolution of the reconstructed image will be dependent on the size of the speckle features. Speckle patterns are nonuniform illumination, which have been previously used in microscopy systems to improve the image resolution beyond the diffraction limit.^191^^,^^202^

According to Zhang et al.,^180^ Fourier patterns can also be employed for the image acquisition process. When compared to Hadamard patterns, Fourier patterns are more efficient, while Hadamard patterns are more resistant to noise. The efficiency of Fourier patterns comes from their ability to concentrate the energy of the image plane. In addition, using multistep Fourier illumination in certain cases can help reduce the number of measurements and therefore the acquisition time.

Rousset et al.^181^ assert that since the compressive sensing reconstruction process based on minimization is computationally expensive, approaches that could yield better reconstruction times are necessary, especially for applications where a fast acquisition time is necessary. Adaptive basis scanning by wavelet prediction has been proposed to allow a more straightforward image recovery than fundamental compressive sensing. This approach involves the use of a wavelet basis such as Haar or LeGall for illumination and is based on predicting the most significant set of patterns to be used for a particular image plane. For the prediction process, a set of initial single-pixel measurements with low-resolution patterns needs to be acquired. This data set can later be used for predicting the significant wavelet coefficients of the image. These coefficients are indexed to specific patterns that will be further used for the main acquisition process. The use of wavelet patterns is justified by the fact that most images can be sparsely characterized in this basis and by the accessibility to inverse wavelet algorithms for image reconstruction.^181^^,^^203^^,^^204^

Another approach that has been proposed to reduce computational time for the image reconstruction process is adaptive compressive sensing imaging. Adaptive approaches have been pursued since the use of basis will often involve measuring background pixels that are not within the sample’s region of interest. If the acquisition patterns can be selected to target the interest area from the sample, the acquisition times may be lowered and the reconstruction process enhanced.

Soldevila et al.^189^ indicated that methods that are based on *a priori* information can be disadvantageous in cases where the imaged sample can unpredictably change. Even though these techniques are beneficial because they define regions of interest on the image plane, it would be better to have a method where no *a priori* information is needed. Adaptive compressive imaging is based on recovering the image under a compressive sensing approach that defines regions of interest during the acquisition process but without the need for *a priori* information. The method involves using sets of masks that will adaptively change depending on the regions of interest on the image plane. The masks can iterate in size depending on the desired resolution. Like other acquisition techniques, it uses inverse wavelet transforms for the image reconstruction process.^189^ The small acquired matrices can then be processed without the need of high computational demand. The image plane is first sampled and delimited by low-resolution masks. Then, a one-level wavelet transform and an edge detection algorithm discard the borderless regions and determine the regions of interest. The high-resolution masks are only applied to these areas. This process can be repeated until the high-resolution masks are only applied to the areas with finer details. Further improvements are still desired on the recognition algorithms to increase the efficiency of the process.

### 4.3 Applications

Single-pixel imaging can be used to monitor fluorescence lifetime.^205^ In the medical imaging field, this new modality has found applications in microscopy^206^ as well as in macroscopic systems.^207^ Pian et al.^187^ reported a single-pixel system that combines hyperspectral detection in the time-domain. A multichannel detector is coupled to a TCSPC unit to perform macroscopic fluorescence lifetime imaging at different detection wavelengths. The structured illumination is produced by DMDs arranged in a transmission or reflection configuration. The system uses the compressive sensing multiplexing scanning methodology and utilizes Hadamard basis as the illumination pattern set. The system has been employed to quantify and image fluorescence lifetime both *in vitro* for tissue-simulating phantoms and *in vivo* for mice. Macroscopic fluorescence lifetime imaging is highly sensible and serves to “unmix” the fluorescence lifetime values of different biomarkers. In addition, the described system^187^ has been used to measure FRET, which describes the interactions of the sample at the molecular level by quantifying the ratio of donor molecules to acceptor molecules and estimating the distance between them.^208^ Figure 7(iii) highlights results obtained from FRET-fluorescence lifetime imaging. Pian et al.^195^ also reported a single-pixel imaging system that can be used for time-resolved hyperspectral tomographic imaging when combining DMD detection and illumination structures in transmission geometry. It has been used to map the concentration of targeted fluorophores.

Finally, Lochocki et al.^188^ proposed that single-pixel imaging systems can be an alternative to the ophthalmoscopes that are necessary to determine a variety of eye illnesses. Even though ophthalmoscopes have been improved throughout the years, they are not fully useful in cases where the ocular structures are opaque. As proposed, a single-pixel camera approach was implemented to image the retina in real-time. The technique was validated in human and artificial eyes to image the fundus of the eye at 15 deg of visual angle. The results obtained from artificial eyes, shown in Fig. 7(ii), highlight the potential of the technique for this application, yet further improvement is needed to correct for eye movements on human subjects.

## 5 Conclusions

The benefits of utilizing spatially structured light projection and collection are manifold, stemming from the ability to create nearly any desired set of spatial input or output configurations in a fast, repeatable manner. This ability helped push the development of spatial frequency-domain photon propagation theory from imaging^22^ to tomographic reconstruction^32^ with contemporaneous development of single-pixel imaging.^177^ Since 2010, diffuse optics has been burgeoning with structured-light techniques taking a lead role in its progress.

Quantitative, noncontact imaging techniques have since been developed for real-time, wide-field acquisition^38^^–^^40^ and have been pushed toward endoscopic implementation.^80^^,^^81^ Models have also been moving toward new speeds and higher accuracy^49^ while becoming robust via subdiffuse sensing.^45^^,^^47^ Many developments in modeling, processing, and instrumentation have helped push efforts toward clinical efficacy, combining SFDI with modalities such as laser speckle,^89^^,^^90^ laser Doppler,^111^ and polarimetric imaging^82^ to accurately predict burn wound severity within 1 h of infliction. Brain imaging with SFDI techniques have demonstrated the potential for identifying Alzheimer’s disease,^84^^,^^112^ its correlated neuronal death,^113^ and for fluorescence-guided tumor resection. ^114^^,^^115^ Oxygenation sensing with SFDI has demonstrated potential in clinical utility for imaging breast cancer,^74^ skin cancer,^56^^,^^116^^,^^209^ skin flap viability,^107^^,^^210^ and has just gained FDA approval for identifying lower limb vascular issues.^75^

DOT has seen new growth due to the flexibility and precision of DMDs. They enable rapid pattern generation for illumination-detection pairs to be sampled fast enough for real-time tomographic sensing^146^ and are sensitive enough for *in vivo* reconstructions with high fidelity.^152^ Rapid acquisition has been coupled with GPU processing^138^ and computationally efficient formulations for reconstructions that take mere minutes on a personal computer.^168^^,^^169^ The use of FRET with structured-light tomography has pushed this limit further and has been demonstrated as a nanoscale proximity assay in small animals.^148^^,^^165^ Endogenous sensing continues to improve with structured light, as real-time tomographic maps of cerebral hemodynamics in a mouse (through the scalp and skull)^171^ are possible and mammogram reconstructions improve.^176^

Single-pixel imaging with a point detector allows for low light level detection and has much greater flexibility than 2-D detection in the chosen optical frequency and bandwidth. Models continue to optimize the selection of illumination patterns for increased image resolution and speed,^181^^,^^189^ while also blending into instrumentation to enable lensless imaging.^190^^,^^191^ Expanding upon and combining these advantages, single-pixel imaging is now capable of hyperspectral, real-time sensing for cancer detection.^207^ In addition, researchers have combined those attributes with fluorescence lifetime imaging and achieve 3-D reconstruction to reach the state-of-the-art in diffuse optical imaging.^187^

The goal of these diffuse optical technologies is to provide quantitative measurements to clinical and preclinical studies. Of these, 2-D imaging techniques such as those based on SFDI have simpler instrumentation and a much simpler model than that of 3-D methods, allowing it to develop more quickly into physiological studies. However, the lack of depth resolution can be a confounding factor for certain layered media,^76^^,^^115^^,^^117^ and so the improvement and utilization of 3-D imagers could greatly enhance our understanding of optical contrast in research and clinical settings. On the other hand, commercially available instruments continually improve, making the 2-D imagers more robust, affordable, and capable every year; impressive studies have been done using even office supply equipment.^70^^,^^72^ As technology simplifies, it is then available for integration with clinical instrumentation such as endoscopes^78^^,^^81^^,^^116^^,^^211^ and begins to provide quantitative imaging *in vivo*, in real-time where was once impossible.
